# UPF1 inhibits the hepatocellular carcinoma progression by targeting long non-coding RNA UCA1

**DOI:** 10.1038/s41598-019-43148-z

**Published:** 2019-04-30

**Authors:** Yongli Zhou, Yandong Li, Na Wang, Xiuying Li, Jianyun Zheng, Liqiao Ge

**Affiliations:** 1 Department of Gastroenterology, The First Affiliated Hospital of Xi’an Medical University, Xi’an, China; 2 Department of Pathology, The First Affiliated Hospital of Xi’an Medical Universtiy, Xi’an, China; 3 General Medical College, Xi’an Medical University, Xi’an, China; 4 Department of Hematology, General Hospital of North China Petroleum Administration Bureau, Renqiu, China; 5 Shanxi University of Traditional Chinese Medicine, Taiyuan, China

**Keywords:** Cancer therapy, Tumour-suppressor proteins

## Abstract

Hepatocellular carcinoma (HCC) is one of the major causes of cancer-related death worldwide. However, the molecular mechanism underlying HCC carcinogenesis remains to be further elucidated. Up-frameshift protein 1 (UPF1) is a RNA/DNA-dependent ATPase and ATP-dependent RNA helicase. Here, we explored the expression and function of UPF1 in HCC. In this study, we demonstrated that UPF1 expression was significantly reduced in hepatocellular carcinoma (HCC) tissues compared with the adjacent normal tissues. And further functional assays revealed that knockdown of UPF1 promoted HCC cells growth and invasion. Furthermore, we found that UPF1 could bind to long non-coding RNA urothelial cancer associated 1 (UCA1) and was negatively correlated with UCA1. UCA1 expression also affected HCC growth and invasion. Knockdown of UCA1 ameliorated the effect of UPF1 knock down on HCC growth and invasion. Knockdown of UPF1 enhances glycolysis in HCC. Taken together, our results provided new insights for finding novel therapeutic targets for hepatocellular carcinoma progression.

## Introduction

Hepatocellular carcinoma (HCC) is one of the major causes of cancer-related death worldwide^1^. HCC results from various types of diseases including liver fibrosis, liver cirrhosis, fatty liver and liver metastasis from other cancers^2–5^. There are so many risk factors leading to the malignant transformation of the hepatocytes^6–8^. The etiology of HCC is so complicated that the details of the molecular mechanisms underlying HCC carcinogenesis remain to be further elucidated.

Up-frameshift protein 1 (UPF1) as a RNA/DNA-dependent ATPase and ATP-dependent RNA helicase is an evolutionarily conserved and ubiquitously expressed phosphoprotein^9^. UPF1 play a key player in nonsense mediated mRNA decay (NMD) and non-NMD RNA degradation^10^. UPF1 associates with a translation termination codon by the translation termination complex during NMD. Moreover, UPF1 also promotes cell progression through G1/S raising the possibility that NMD promotes the decay of mRNAs encoding inhibitory proteins that block progression through this stage of the cell cycle^11^. Recent study also found that the human RNA surveillance factor UPF1 regulates tumorigenesis^12^.

In the present study, we demonstrated that UPF1 expression was significantly reduced in hepatocellular carcinoma (HCC) tissues compared with the adjacent normal tissues. And further functional assays revealed that knockdown of UPF1 promoted HCC cells growth and invasion. Furthermore, we found that UPF1 could bind to long non-coding RNA UCA1 and negatively correlated with UCA1. UCA1 expression also affected HCC growth and invasion. Knockdown of UCA1 ameliorated the effect of UPF1 knock down on HCC growth and invasion. Knockdown of UPF1 enhances glycolysis in HCC. Taken together, our results provided new insights for finding novel therapeutic targets for hepatocellular carcinoma progression.

## Materials and Methods

### Clinical samples

Fifty pairs of liver cancer and adjacent non-tumor samples were collected from hepatocellular cancer patients and stored in liquid nitrogen before use. The written informed consent was obtained from all patients before their inclusion in this study. The Medical Ethics Committee of the Affiliated Hospital of Xi’an Medical University approved ethical approval for this study. All methods were performed in accordance with the relevant guidelines and regulations of Medical Ethics Committee of the Affiliated Hospital of Xi’an Medical University.

### Cell culture

Human hepatocellular carcinoma Huh7 and HeG2 cells were cultured with RPMI 1640 medium supplemented with 10% fetal bovine serum (Gibco, Grand Island, NY, USA), 100 U/mL penicillin, and 100 μg/mL streptomycin (Gibco) in an atmosphere of 37 °C, 5% CO2.

### Immunohistochemistry (IHC)

For IHC, the procedures were performed as the previous study^13^. Simply, the paraffin-embedded sections were deparaffinized, rehydrated. The sections were incubated in anti-UPF1 antibody (Sigma-Aldrich, St. Louis, MO, USA) with a dilution of 1:200 overnight at 4 °C and then incubated with secondary antibody accordingly. Finally, a peroxidase substrate was added and incubated until desired stain intensity developed. Tissue sections were mounted on slides using mounting medium.

### Quantitative real-time reverse transcription-PCR

Total RNA from clinical samples was extracted using Trizol reagent (Invitrogen, Carlsbad, CA, USA) and was reverse-transcribed using a PrimeScriptRT reagent Kit (Promega, Madison, WI, USA) according to the manufacturer’s instruction. In brief, quantitative real-time reverse transcription -PCR was then performed using SYBR Green Master Mix (Thermo Fisher Scientific, Waltham, MA USA) and ABI7900HT Real Time PCR system (Applied Biosystems, Foster City, CA, USA). Glyceraldehyde-3-phosphate dehydrogenase (GAPDH) was used as internal control. The primers used are as follows GAPDH (5′-CCATGTTCGTCATGGGTGTGAACCA-3′ and 5′-GCCAGTAGAGGCAGGGATGATGTTG-3′), UPF1 (5′-ACCGACTTTACTCTTCCTAGCC-3′ and 5′-AGGTCCTTCGTGTAATAGGTGTC-3′) and UCA1 (5′- ATGCTTCCATGCAACACTCCT-3′ and 5′-GATTTTTTGTTTTGGGTGTGG-3′). The relative expression levels of each gene were calculated and determined using the 2^−ΔΔct^ method.

### siRNA synthesis and cell transfection

siRNAs targeting UPF1 or UCA1 was synthesized by the Ribobio (Guangzhou, China). The negative control siRNA was also purchased from Riobobio.

Cells were transfected using Lipofectamine 2000 transfection reagent (Thermo Fisher Scientific, Waltham, MA USA) according to the manufacturer’s instructions. At 48 hours after transfection, the cells were harvested, and the extracts were for further study. Plasmids were also used to transfect as the same above method.

### Methyl-thiazolyl-tetrazolium (MTT) assay

Cells were plated onto 96-well plates before incubation overnight. The medium was exchanged every 24 hours. The MTT (Sigma-Aldrich, St. Louis, MO, USA) was added to the cell supernatant, and removed after four hours at 37 °C. Then, 200 µl DMSO was added to each well and shaken for 15 minutes. The optical density (OD) value was measured at 490 nm by an enzyme micro-plate reader. Each experiment was repeated three times.

### Western blotting

Protein concentrations of cell lysate were measured using a standard BCA assay according to the manufacturer’s instructions. Proteins were separated in SDS–PAGE (10%) and then transferred to nitrocellulose membranes at 4 °C. Membranes were blocked with 5% nonfat dry milk in TBST for 1 hour, and then incubated overnight at 4 °C with the antibodies for UPF1 or GAPDH (Cell Signaling, Danvers, MA). After washing with TBST, membranes were incubated with the secondary antibody for 1 hour at room temperature. The signals were developed with the ECL kit.

### Colony formation

Firstly, cells were plated in six-well plates (5 × 10^3^ cells per well) before incubation overnight, and then cultured in RPMI 1640 medium for 2 weeks after transfection. After the cells were washed with PBS twice, the cells were stained with 0.1% crystal violet. Finally the colonies with more than 50 cells were counted.

### Transwell invasion assay

The invasion assays were performed by a Transwell chamber, according to the manufacturer’s protocol. Briefly, the membranes of each upper chamber of an insert were coated with Matrigel Basement Membrane Matrix (BD Biosciences, Bedford, MA, USA) and then incubated for 5 h at 37 °C. Cells were seeded in the upper chamber with serum-free media, and the lower chamber was loaded with 700 μl of complete medium. And then the cells were incubated for 24 h, washed, fixed, and then stained with crystal violet before counting under a microscope.

### RNA stability assay

Huh7 and HepG2 cells transfected with siRNA targeting for UPF1/UCA1 or control were incubated with 5 μg/ml of Actinomycin D (Sigma-Aldrich, St. Louis, MO, USA) in the medium. Total RNA was harvested at the indicated time and then mRNA expression level was evaluated by real time PCR. The mRNA half-life was detected before and after adding Actinomycin D.

### RNA immunoprecipitation assay

RNA immunoprecipitation (RIP) were carried out as described previously^14^. In brief, cells were harvested using a mixed buffer on ice for 20 min. Then nuclei were pelleted by centrifugation at 2,500 g for 15 min and then nuclear pellets were resuspended in RIP buffer. Nuclear membrane and debris were pelleted by centrifugation. Antibody to rabbit IgG or UPF1 (Cell Signaling, Danvers, MA) was added to supernatant together with protein G beads (Thermo Fisher Scientific, Waltham, MA USA) and incubated overnight at 4 °C. Then co-precipitated RNAs were isolated and qRT-PCR for UCA1 was conducted.

### Glucose consumption and lactate production assay

To measure the levels of glucose and lactate, cell supernatants were collected and detected by a glucose and lactate assay kit (BioVision, Milpitas, CA, USA) according to the manufacturer’s instructions.

### Statistical analysis

The statistical analyses were performed by SPSS 16 software. All the experiments were performed three times, and the data were presented as the mean ± SD. The results were analyzed using one-way ANOVA and Student’s t-test. *P* < 0.05 was considered statistically significant.

## Results

### UPF1 expression was decreased in HCC

**Figure 1:**
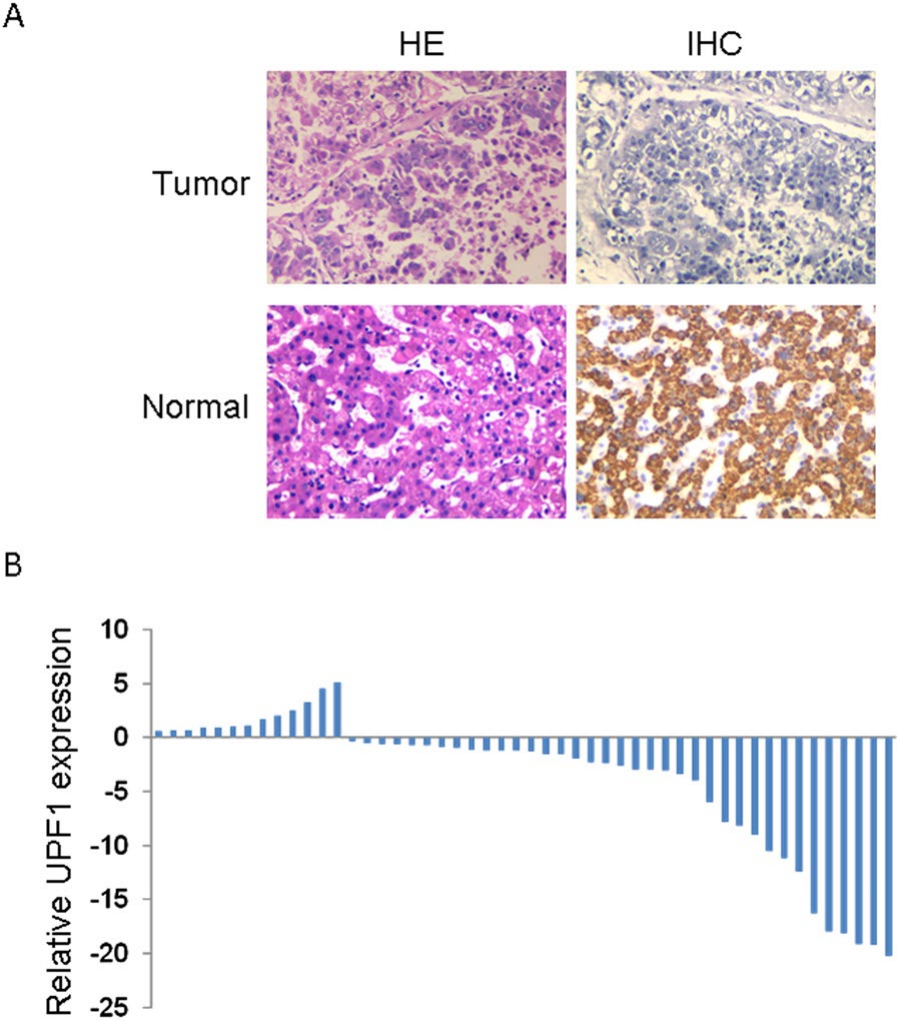
UPF1 expression in human HCC tissues. (**A**) UPF1 expression was detected in fifty pairs of human Hepatocellular Carcinoma and adjacent normal samples by IHC (200×). The expression of UPF1 was decreased in HCC tissues. (**B**) Relative expression (log2-transformed value) of UPF1 in HCC tissue (n = 50) compared with corresponding non-tumor tissue (n = 50). UPF1 expression was detected by real time PCR and normalized to GAPDH expression. The results are presented as the fold-change in tumor tissues relative to normal ones.

To investigate the expression and clinical significances of UPF1 in Hepatocellular Carcinoma (HCC), we first measured UPF1 expression in 50 cancer tissues and paired adjacent noncancerous HCC tissues. As shown in Fig. 1A, IHC illustrated that the expression of UPF1 was decreased in HCC tissues compared with adjacent non-tumor tissues. We next detected the expression of UPF1 in these tissues by real time PCR and also found the low expression of UPF1 in HCC tissues (Fig. 1B). Fifty cases were divided into two groups: a high UPF1 expression group (above the median UPF1 expression) and a low UPF1 expression group (below the median). As shown in Supplementary Table S1, the correlation regression analysis revealed that the expression of UPF1 was associated with tumor size and lymph node metastasis. These indicated that UPF1 was involved in HCC and might play an important role in tumor progression.

### Silencing of UPF1 promoted HCC cells growth and invasion

**Figure 2:**
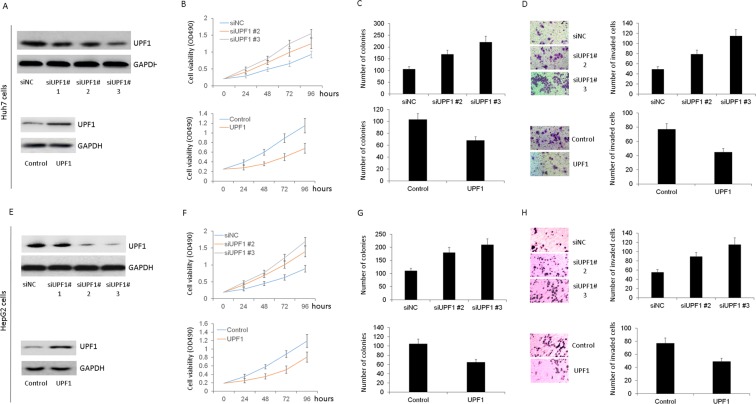
Silencing of UPF1 promoted HCC cells growth and invasion. Western blot confirmed the knockdown efficiency or overexpression of UPF1 in Huh7 (**A**) and HepG2 cells (**E**). (**B** and **F**) MTT assay was used to test the cell proliferation in Huh7 and HepG2 cells. (**C** and **G**) Colony formation assay was also used to test effect of UPF1 on cell growth. (**D** and **H**) Knockdown of UPF1 significantly increased cell invasion rates (upper panel) and overexpression of UPF1 decreased the cell invasion ability (lower panel) by transwell assay.

To further investigate the roles of UPF1 in HCC, we silenced UPF1 expression by transfecting specific siRNAs targeting UPF1 into HCC Huh7 and HepG2 cells. The transfection efficiently decreased the expression of UPF1 valuated by western blotting (Fig. 2A,E). Among three siRNAs the second and third ones were more efficient, and therefore the number #2 and #3 were used to conduct the subsequent experiments. Firstly, MTT assay was carried out in HCC cell lines. After expression of UPF1 was downregulated in Huh7 and HepG2 cells, the cell proliferation increased (Fig. 2B,F). Colony formation assay was also used to test effect of UPF1 on cell growth. Figure 2C,G showed that the colony-forming ability of cells was enhanced after knockdown of UPF1. We next assessed the effects of UPF1 on cell invasion in Huh7 and HepG2 cells. As shown in Fig. 2D,H, knockdown of UPF1 significantly increased cell invasion rates, moreover, the overexpression of UPF1 decreased cell invasion ability. These findings suggested that silencing of UPF1 promoted HCC cells growth and invasion.

### UPF1 could bind to long non-coding RNA UCA1

Recently, there are several reports that many lncRNAs participate in molecular regulation pathways through their interactions with proteins^15^. Online software starBase v2.0 database was used to identify lncRNAs that potentially combine with UPF1. We found that UCA1 was able to bind to UPF1.

**Figure 3:**
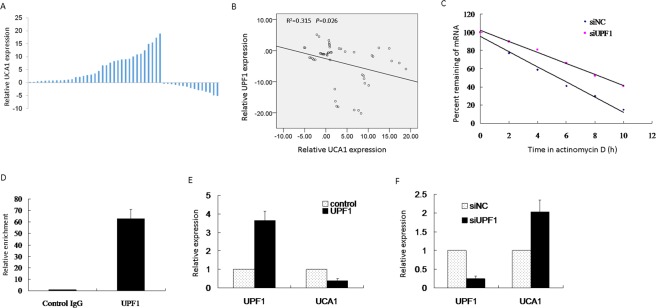
Long non-coding RNA UCA1 was able to bind to UPF1. (**A**) UCA1 expression was detected by real time PCR and normalized to GAPDH expression. (**B**) Bivariate correlation between UPF1 and UCA1 RNA expression level in 50 HCC tissues. (**C**) Cells were treated with Actinomycin D for the indicated time and UCA1 RNA level was evaluated by real time PCR. (**D**) RIP assay illustrated the binding between UCA1 and UPF1. (**E**) The overexpression of UPF1 decreased the expression of UCA1. UPF1 plasmid was transfected into Huh7 cells. (**F**) The knockdown of UPF1 in Huh7 cells increased the expression of UCA1.

Firstly, the expression level of UCA1 was studied in HCC by real time PCR. Results showed that the expression of UCA1 was higher in HCC than adjacent noncancerous HCC tissues (Fig. 3A). To further explore the relationship between UPF1 and UCA1 in HCC, we analyzed their expression levels in HCC tissues. Figure 3B showed that the RNA expression level of UPF1 and UCA1 in the paired tissues was negative associated by RT-PCR. RNA stability of UCA1 was further tested in HCC cells with UPF1 knockdown. As shown in Fig. 3C, the decay rate of UCA1 was increased in Huh7 after knockdown of UPF1. Next, we analyze the binding between UCA1 and UPF1 by RIP, and found that UPF1 specially combined with UCA1 (Fig. 3D). We also transfected UPF1 expressed plasmid into Huh7 cells. The overexpression of UPF1 decreased the expression of UCA1 (Fig. 3E). While, the knockdown of UPF1 in Huh7 cells increased the expression of UCA1 (Fig. 3F). The above data hinted that UPF1 combined with UCA1 and could potentially take part in HCC progression.

### Effect of UCA1 expression on HCC growth and invasion

**Figure 4:**
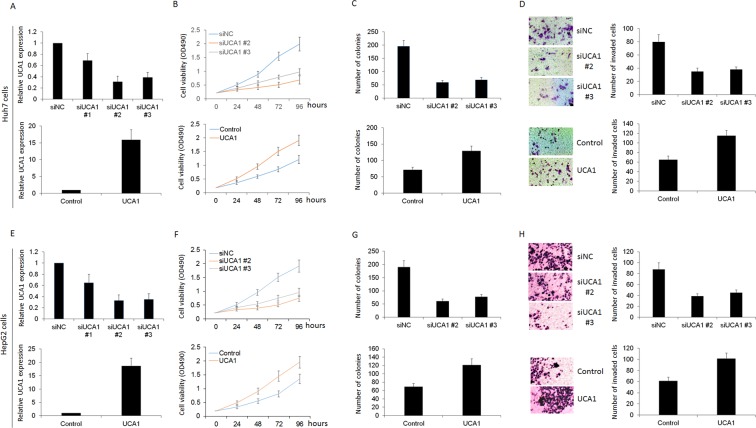
Effect of UCA1 expression on HCC growth and invasion. (**A** and **E**) The expression of UCA1 in Huh7 and HepG2 cells was knocked down or overexpression to study the function of UCA1. MTT was used to analyze cell proliferation in Huh7 (**B**) and HepG2 (**F**) cells. (**C** and **G**) Colony formation assay showed that the knockdown or overexpression of UCA1 reduced or enhanced the Huh7 cell and HepG2 growth. We also evaluated the effects of UCA1 on cell invasion in Huh7 (**D**) and HepG2 (**H**) cells by transwell assay.

We also analyzed the correlation between UCA1 expression and clinicopathological characteristics of these 50 HCC patients. Fifty cases were divided into two groups: a high UCA1 expression group (above the median UCA1 expression) and a low UCA1 expression group (below the median). As shown in Supplementary Table S2, the correlation regression analysis revealed that high expression of UCA1 was associated with large tumor size (*p* = 0.032) and lymph node metastasis (*p* = 0.033), which implied that UCA1 may be involved in the growth and invasion of HCC. Then the expression of UCA1 in Huh7 and HepG2 cells was knocked down to study the function of UCA1 and the siRNA number #2 and #3 were more efficient target for UCA1 to conduct subsequent experiments (Fig. 4A,E). To examine the effects of UPF1 on HCC growth, MTT assay have been done. As shown in Fig. 4B,F, knockdown of UCA1 decreased the cell progression of HCC cells. In addition, colony formation assay showed that the knockdown of UCA1 reduced the Huh7 and HepG2 cell growth (Fig. 4C,G). We next evaluated the effects of UCA1 on cell invasion by transwell assay. After down-regulation of UCA1 expression, the invasion capacity was decreased, and then overexpression of UCA1 increased cell invasion (Fig. 4D,H). All these data suggested that UCA1 may be involved in HCC growth and invasion.

### Knockdown of UCA1 ameliorated the effect of UPF1 knock down on HCC growth and invasion

**Figure 5:**
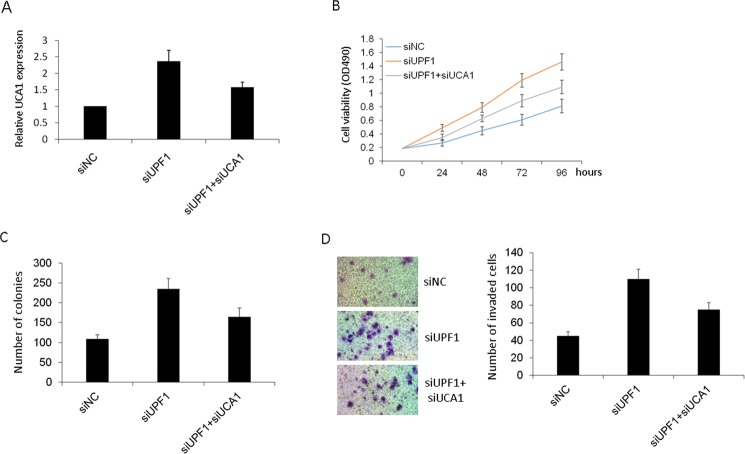
Knockdown of UCA1 ameliorated the effect of UPF1 knock down on HCC growth and invasion. (**A**) The expression of UCA1 in Huh7 cells was investigated. (**B**) MTT was used to analyze cell proliferation. (**C**) Colony formation assay. (**D**) The cell invasion was analyzed by transwell assay.

To further investigate the relationship between UPF1 and UCA1, the siRNA for UCA1 was transfected into HCC after knockdown of UPF1. Firstly, real time PCR showed that the knockdown of UCA1 decreased the expression of UCA1 after UPF1 knock down increasing UCA1 (Fig. 5A). MTT assay showed that the knockdown of UPF1 increased the HCC proliferation, while UCA1 knock down decreased the cell proliferation ability (Fig. 5B). Next colony formation assay was used to further study cell growth, which was also weakened after the knockdown of UCA1 (Fig. 5C). Finally, we observed that cell invasion ability was decreased after the knockdown of UCA1 by transwell assay (Fig. 5D). These suggested that knockdown of UCA1 ameliorated the effect of UPF1 knock down on HCC growth and invasion.

### Knockdown of UPF1 enhances glycolysis in HCC

**Figure 6:**
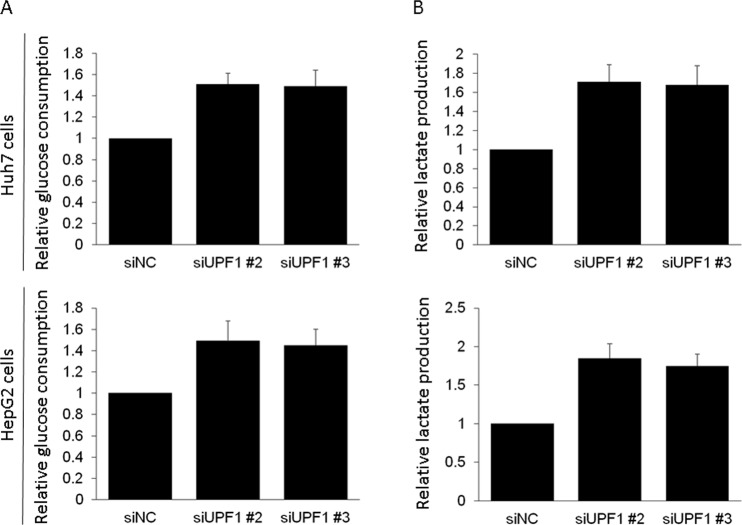
Knockdown of UPF1 enhances glycolysis in HCC. (**A**) Glucose consumption was analyzed in HCC. (**B**) Lactate production was detected in HCC.

Usually tumor cells exhibit a higher rate of glucose metabolism compared with normal cells^16^. It is reported that UCA1 was involved in tumor cell glycolysis^17^. Next, we investigated the changes in glycolysis in HCC with knockdown of UPF1. The results showed that knockdown of UPF1 increased the rates of glucose consumption in HCC (Fig. 6A). We also found that UPF1 knockdown increased the rates of lactate production in HCC (Fig. 6B). These suggested that knockdown of UPF1 enhances glycolysis in HCC.

## Discussion

Here, we reported that the correlation between UPF1 and HCC. IHC and real time PCR showed that UPF1 expression was decreased in HCC and might play an important role in tumor progression. We also identified the function of UPF1 in the HCC cells by applying loss-of-function approaches. Our data clearly demonstrated that knockdown of UPF1 promoted HCC cells growth and invasion.

Up-frameshift (Upf) complex facilitates the degradation of aberrant mRNAs^18,19^. UPF1 has been characterized as an essential factor for nonsense-mediated mRNA decay (NMD)^20,21^. UPF1 is essential for embryonic development and survival^22,23^. The loss of UPF1 function inhibits cell growth and induces apoptosis in Drosophila melanogaster^24^. UPF1 also takes part in cancer progression. UPF1 is a potential modulator of MALAT1 and that UPF1/MALAT1 pathway could be a therapeutic target for gastric cancer^25^. UPF1 was expressed at lower levels in human lung adenocarcinoma tissues than in normal lung tissues, thereby raising the possibility that NMD may be downregulated to permit lung adenocarcinoma oncogenesis^26^. Our results were consistent with these previous reports that UPF1 was a tumor suppressor.

Long non-coding RNAs (lncRNAs) are defined as those >200 nucleotides (nt). LncRNAs are coming from the “noisy region” of the genome as a new source of biomarkers that could characterize disease recurrence and progression^27^. LncRNAs function in various aspects of cell biology has focused increasing attention on their potential to contribute towards tumor development^28–30^. Urothelial cancer associated 1 (UCA1) is a long noncoding RNA having aberrant expression in embryogenesis and a broad range of cancer tissues and cells^31,32^. Especially, UCA1 plays oncogenic roles in tumor growth and metastasis^33,34^. However, the molecular mechanism of UCA1 in cancer progression remains incompletely understood. In this study, UPF1 could bind to UCA1 and the correlation between UPF1 and UCA1 was negative. Knockdown of cellular UCA1 significantly decreased the cell growth and invasion.

In conclusion, our present study uncovers a novel UPF1-mediated mechanism of cell growth and invasion by targeting long non-coding RNA UCA1 in HCC cells. Our results indicated that UPF1/UCA1 played an important role during HCC tumorigenesis and may serve as a putative target for HCC diagnosis and therapy.

## Supplementary information

Supplementary Information
