# Supplementary material for: UPF1 inhibits the hepatocellular carcinoma progression by targeting long non-coding RNA UCA1
Source: Sci Rep. 2019 Apr 30;9:6652. doi: 10.1038/s41598-019-43148-z (PMC6491801; doi:10.1038/s41598-019-43148-z)
Supplement: Supplementary file 1 — Supplementary Information [file 41598_2019_43148_MOESM1_ESM.pdf]

UPF1 inhibits the hepatocellular carcinoma progression by targeting long non-coding RNA UCA1

Yongli Zhou[1,3], Yandong Li[2,3], Na Wang[4], Xiuying Li[5], Jianyun Zheng[2,3], Liqiao Ge[1,3]

[1]Department of Gastroenterology, The First Affiliated Hospital of Xi'an Medical University, Xi'an, China

[2]Department of Pathology, The First Affiliated Hospital of Xi'an Medical Universtiy, Xi'an, China

[3]General Medical College, Xi'an Medical University, Xi'an, China

[4]Shanxi University of Traditional Chinese Medicine, Taiyuan, China

[5]Shanxi University of Traditional Chinese Medicine, Taiyuan, China

Running title: UPF1 inhibits HCC by UCA1

***Correspondence author:** Dr. Yandong Li, Department of Pathology, The First Affiliated Hospital of Xi'an Medical University, No.48 Fenggao West Road, Xi'an 710077, China

Email: liyandongzhongnan@163.com

Disclosure of conflict of interest

None.

Supplementary Table S1. Relationship between expression of UPF1 and clinicopathological parameters in hepatocellular carcinoma

Variable	Cases	High expression (%)	Low expression (%)	χ^2	*P* value
Gender					
Male	28	11 (39%)	17 (61%)	0.007	0.935
Female	22	8 (36%)	14 (64%)		
Age					
≤60	20	7 (35%)	13 (65%)	0.004	0.953
>60	30	12 (40%)	18 (60%)		
Differentiation					
High+ Median	27	15 (56%)	12 (44%)	6.144	0.013
Low	23	4 (17%)	19 (83%)		
Stage					
I	15	6 (40%)	9 (60%)	0.028	0.867
II	16	6 (38%)	10 (62%)		
Ⅲ	19	7 (37%)	12 (63%)		
LN metastasis					
Positive	31	8 (26%)	23 (74%)	3.876	0.049
Negative	19	11 (58%)	8 (42%)		

Supplementary Table S2. Relationship between expression of UCA1 and clinicopathological parameters in hepatocellular carcinoma

Variable	Cases	High expression (%)	Low expression (%)	χ^2	*P* value
Gender					
Male	28	15 (54%)	13 (46%)	0.004	0.945
Female	22	12 (55%)	10 (45%)		
Age					
≤60	20	11 (55%)	9 (45%)	0.030	0.861
>60	30	17 (57%)	13 (43%)		
Differentiation					
High+ Median	27	12 (44%)	15 (56%)	4.593	0.032
Low	23	15 (65%)	8 (35%)		
Stage					
I	15	8 (53%)	7 (47%)	0.093	0.760
II	16	9 (60%)	6 (40%)		
III	19	10 (53%)	9 (47%)		
LN metastasis					
Positive	31	25 (81%)	6 (19%)	4.563	0.033
Negative	19	9 (47%)	10 (53%)		

Figure S1

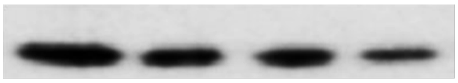

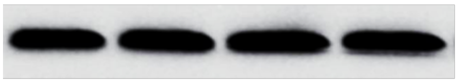

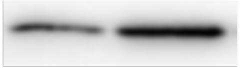

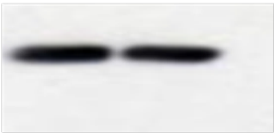

Figure S1: Full-length gel shown in Figure 2A.

Figure S2

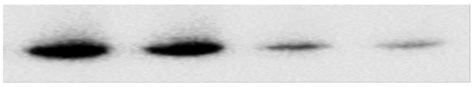

UPF1

siNC siUPF1#1 siUPF1#2 siUPF1#3

HepG2 cells

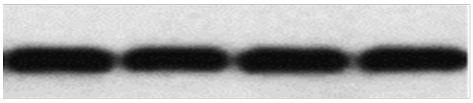

GAPDH

siNC siUPF1#1 siUPF1#2 siUPF1#3

HepG2 cells

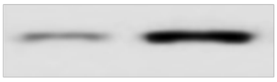

UPF1

Control UPF1

HepG2 cells

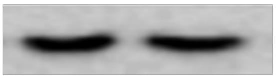

GAPDH

Control UPF1

HepG2 cells

Figure S2: Full-length gel shown in Figure 2B.
